# A review of the primary types of the Hawaiian stag beetle genus *Apterocyclus* Waterhouse (Coleoptera, Lucanidae, Lucaninae), with the description of a new species

**DOI:** 10.3897/zookeys.433.8022

**Published:** 2014-08-13

**Authors:** M.J. Paulsen, David C. Hawks

**Affiliations:** 1 Systematics Research Collections, University of Nebraska State Museum, W436 Nebraska Hall, Lincoln, NE 68588-0546 USA; 2 Department of Entomology, University of California- Riverside, Riverside, CA 92521 USA

**Keywords:** Systematics, Scarabaeoidea, Hawaii, endemic, taxonomic revision

## Abstract

The species of the Hawaiian stag beetle genus *Apterocyclus* Waterhouse (Coleoptera: Lucanidae) are reviewed following an examination of all primary types. Although the continued existence of the species is unknown and some possibly are extinct there are five recently extant species, including one species that is described here as new. The holotypes for all available names are pictured, and synonymies discussed and updated. Lectotypes are designated for *Apterocyclus honoluluensis* Waterhouse and *A. munroi* Sharp. A key to species and a revised catalog for the genus are provided.

## Introduction

The Hawaiian endemic genus *Apterocyclus* Waterhouse (Coleoptera: Lucanidae: Lucaninae) contains the only species of scarabaeoid beetles native to the Hawaiian Islands (Howden 2008). They represent the most isolated genus of stag beetle, being at least 3750 km distant from any other genus. As with several other island-endemic genera of Lucanidae (*Agnus* Burmeister, *Amneidus* Coquerel, *Bomansius* Lacroix, *Microlucanus* Bomans & Bartolozzi, *Vinsonella* Arrow), species of *Apterocyclus* are flightless. Previous workers have struggled with the high degree of morphological variability in the genus (Sharp and Scott 1908, Van Dyke 1922), which may be a result of limited gene flow in variably isolated populations due to flightlessness and the rugged terrain of Kaua’i.

Waterhouse (1871) described the genus and its first species as *Apterocyclus honoluluensis* in honor of the origin of the shipment (Honolulu, Oahu), not the actual locality. This has become a famous case of misapplied geography in nomenclature; the genus is only found on the island of Kaua’i. Sharp attempted to address the high degree of morphological variation in the genus by proposing six additional species, although he suffered from a limited number of specimens to study (Sharp and Scott 1908). Van Dyke (1922) examined over 130 specimens, and the apparent morphological plasticity led him to synonymize Sharp’s names and place all *Apterocyclus* species together again under *Apterocyclus honoluluensis*. Following Van Dyke, most of Sharp’s species have been considered synonyms by subsequent authors. Of Sharp’s six available names, only *Apterocyclus waterhousei* Sharp is frequently recognized as distinct (e.g., Benesh 1960, Maes 1992, Jameson et al. 2009).

In 2011–2012, one of us (MJP) assisted the Natural History Museum, London, UK, in the location and repatriation of the seven Waterhouse and Sharp primary types of *Apterocyclus* that had been out on a derelict loan forgotten by the borrower for more than a dozen years. After the types were returned and once again available for study we were able to reexamine this problematic genus and form species hypotheses based on morphology. The most useful characters studied included the shape of the protibia, mandibles, ocular canthus, and presence or absence of elytral setae. The male genitalia of all species are more or less uniform and not useful for delineating species. Even with the variability within species and lack of distinct male genitalia, we have identified five species that are supported by clear and consistent morphological differences. Although we believe Van Dyke’s (1922) work on other lucanids to be more competent than his near contemporaries, we found that he was too conservative when he synonymized some of Sharp’s species under *Apterocyclus honoluluensis*. We readdress the synonymies and discuss the characters distinguishing five species below, including the description of one new species. A key is provided for the genus that is functional for all known specimens, although the females of three species remain unknown.

## Materials and methods

Specimens of the genus are found in relatively few collections. We examined 143 specimens from or deposited in the following collections: Bernice P. Bishop Museum, Honolulu, HI, USA (BPBM), California Academy of Sciences, San Francisco, CA, USA (CASC); Canadian Museum of Nature Collection, Ottawa, ON, Canada (CMNC); M.J. Paulsen Collection, Lincoln, NE, USA (MJPC); Montana Entomology Collection, Bozeman, MT, USA (MEC); and the Natural History Museum, London, UK (NHM).

## Results

Unfortunately, the number of *Apterocyclus* specimens available for study in public collections has not increased greatly since Van Dyke’s study, and this may be due to the increased rarity of the species due to habitat loss and ecological change. Based on subfossils from caves at low elevation studied by Nick Porch (Deakin University, Australia), Elias (2010) considered it likely that ‘most’ of the *Apterocyclus* taxa had become extinct after the arrival of people, surviving only above 800-900m today. Indeed, very few specimens from the last half-century have been placed in institutional collections. For *Apterocyclus honoluluensis* these include the 1972 CMNC pair reported by Howden (2008), over 40 specimens examined from 1975–1979 (BPBM, MEC), and one from 2005 (MEC). The two known specimens of *Apterocyclus kawaii* sp. n., are from 1978 (BPBM) and 1996 (Abbott and Petr 1997). The report in Jameson et al. (2009) of adult and larval specimens of *Apterocyclus waterhousei* collected in 2004 is doubly incorrect; the single adult specimen associated with the larvae from the attempted rearing (MEC) is *Apterocyclus honoluluensis*, and labels indicate that the larvae were collected in 2005, not 2004. While no recent specimens of *Apterocyclus waterhousei* were located in institutional collections, as with *Apterocyclus honoluluensis* the species has been found recently by amateur collectors. For example, the two specimens of *Apterocyclus waterhousei* shown in Fujita (2010) are reported from 1997 and 1998. Because this means that only three of the five species have been confirmed to be extant during the last fifty years and with few specimens available for study of any species, the present distributions and conservation needs of all five species warrant urgent study. The following treatment is presented to clarify the taxonomy of the genus in preparation for such further studies, including a molecular study of the geographical origins of the genus by identifying their most closely related genera among world Lucanidae.

## Taxonomic treatment

### Apterocyclus honoluluensis

Taxon classificationAnimaliaColeopteraLucanidae

Waterhouse, 1871

Figs 1–4 , 12 , 17

Apterocyclus honoluluensis Waterhouse 1871: 315. Type material: Lectotype male of *A. honoluluensis* (NHM) labeled: a) handwritten, “Kauai/ 4000 ft. / W.H. Pease / 71-29”; b) handwritten, “Apterocyclus / honoluluensis / ♀ (sic) Type C. Waterh. “ c) red-bordered circular label “Type”; d) red paper, “*Apterocyclus* / *honoluluensis* ♂ / Waterhouse, 1871 / LECTOTYPE / des. M.J. Paulsen”; e) “*Apterocyclus* / *honoluluensis* / Waterhouse, 1871 / det. M.J. Paulsen 2012”. Paralectotype of *A. honoluluensis* (NHM) = holotype of *A. deceptor*, below.
Apterocyclus deceptor Sharp in Sharp and Scott 1908: 405, synonymy confirmed. Type material: Holotype male of *A. deceptor* labeled: a) handwritten, “Kauai/ 4000 ft. / W.H. Pease / 71-29”; b) handwritten, “Apterocyclus / honoluluensis / ♂ Type C. Waterh. “ c) red-bordered circular label “Type”; d) handwritten, “*Apterocyclus* / *deceptor* / Type D.S.”; e) yellow paper, “*Apterocyclus* / *honoluluensis* ♂ / Waterhouse, 1871 / PARALECTOTYPE / des. M.J. Paulsen”; f) red paper, “*Apterocyclus* / *deceptor* ♂ /Sharp, 1908 / HOLOTYPE / des. M.J. Paulsen”; g) “*Apterocyclus* / *honoluluensis* / Waterhouse, 1871 / det. M.J. Paulsen 2012”.
Apterocyclus feminalis Sharp in Sharp and Scott 1908: 405, synonymy confirmed. Type material: Holotype female of *A. feminalis* (NHM) labeled: a) handwritten, on cork “*Platycerus feminalis*, / typ. D.S. / Kauai, 4000 ft. VII-’96 / Perkins”; b) “Sandwich Is. / 1912-215”; c) red-bordered circular label “Type”; d) red paper, “*Apterocyclus* / *feminalis* ♀ /Sharp, 1908 / HOLOTYPE / des. M.J. Paulsen”; e) “*Apterocyclus* / *honoluluensis* / Waterhouse, 1871 / det. M.J. Paulsen 2012”.
Apterocyclus varians Sharp in Sharp and Scott 1908: 404, synonymy confirmed. Type material: Holotype male of *A. varians* (NHM) labeled: a) handwritten, on cork “Mts: Waimea Kauai / 5000 ft. Perkins IV.1894 / *A. varians* / Type D.S.”; b) red-bordered circular label “Type”; c) “Sandwich Is. / 1912-215”; d) handwritten, “*varians* Sharp”; e) red paper, “*Apterocyclus* / *varians* /Sharp, 1908 / HOLOTYPE / des. M.J. Paulsen”; g) “*Apterocyclus* / *honoluluensis* / Waterhouse, 1871 / det. M.J. Paulsen 2012”.

#### Diagnosis.

**Figures 1–4.**
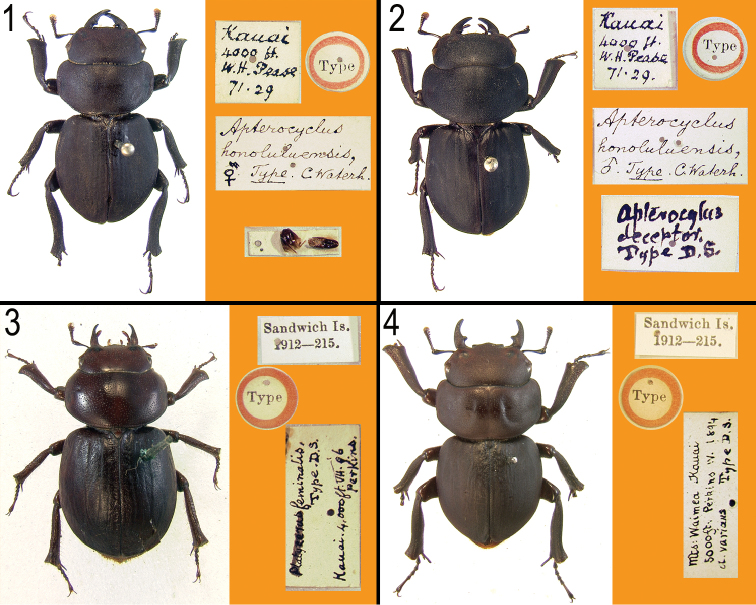
Primary types of *Apterocyclus honoluluensis* and its synonyms, with labels. **1** *Apterocyclus honoluluensis* Waterhouse, lectotype **2** *Apterocyclus deceptor* Sharp, holotype **3** *Apterocyclus feminalis* Sharp, holotype **4** *Apterocyclus varians* Sharp, holotype.

This smallest species (14–17 mm, rarely up to 21 mm) can be readily distinguished by the presence of a short but distinct ocular canthus (Fig. 1), which is not present in the remaining four species. Otherwise, as lamented by Van Dyke (1922), there is great morphological variability in the species. In most specimens the protibia lacks any external teeth proximal to the apex. A few specimens of each sex exhibit a single weak external tooth on the protibia, unlike the majority of specimens for which the entire external margin of the protibiae is otherwise a useful diagnostic character. Based on the specimens studied there is also pronounced variability in males in surface sculpture (shiny vs. alutaceous) and mandibular shape. Some male specimens have mandibles with traces of additional internal teeth. Females are more strongly shiny and can be distinguished by their mandibles completely lacking internal teeth and their more spherical abdomens. Without additional collecting to better define the distribution and pinpoint potentially isolated populations it is not possible to determine if the morphological variation present is actually taxonomically important.

#### Distribution.

This was historically, and has been recently, the most commonly collected species. It is known from various localities in Kōke’e State Park.

#### Remarks.

Waterhouse (1871) examined two specimens collected by Harper Pease in the “Mountains of Kanoi”, which may be a mistranslation of Kaua’i as we can find no locality with that name. Waterhouse considered the specimens to be a male and female. As indicated by Sharp (1908), both specimens actually are males. Waterhouse illustrated the “female” and Sharp (1908) considered that specimen to be the type of *Apterocyclus honoluluensis* (Fig. 1). We designate that specimen here as the lectotype of *Apterocyclus honoluluensis*, in agreement with ICZN recommendation 74B. The other specimen becomes a paralectotype of *Apterocyclus honoluluensis*, and it also is the holotype of *Apterocyclus deceptor* Sharp (Fig. 2). The holotypes of *Apterocyclus deceptor*, *Apterocyclus feminalis* (Fig. 3), and *Apterocyclus varians* (Fig. 4) are conspecific with *Apterocyclus honoluluensis*, and these synonymies proposed by Van Dyke (1922) are reconfirmed.

Van Dyke (1922) indicated that the larvae were formerly abundant in the soil, when hundreds could be encountered while digging. Mizunuma (2000) reported that the larvae feed in soil that contains the decomposing trunks of the leguminous tree *Acacia koa* A. Gray (Fabaceae). Mizunuma (2000) also described the larvae of *Apterocyclus honoluluensis* as being sensitive to, and killed by, high temperature. In 1972, Howden found evidence of heavy predation on adults of *Apterocyclus honoluluensis* by introduced mice, as might be expected for a flightless insect exposed to a novel terrestrial omnivore (Howden 2008).

### Apterocyclus munroi

Taxon classificationAnimaliaColeopteraLucanidae

Sharp, 1908

Figs 5–6 , 14 , 19

Apterocyclus munroi Sharp in Sharp and Scott 1908: 403, **revised status.** Type material: Lectotype male of *A. munroi* (NHM) labeled: a) handwritten, on cork “Type D.S. / *Apterocyclus munroi* / Kaula, Kauai. VII.1897 / G.C. Munro; b) red-bordered circular label “Type / H.T.”; c) “Sharp Coll. / 1905:313”; c) red paper, “*Apterocyclus* / *munroi* ♂ /Sharp, 1908 / LECTOTYPE / des. M.J. Paulsen”; g) “*Apterocyclus* / *munroi* / Sharp, 1908 / det. M.J. Paulsen 2012”. Paralectotype male (NHM) labeled: a) handwritten, on cork “*Apterocyclus* / *munroi* / 2nd Typ. D.S.”; b) “Sharp Coll. / 1905-313”; c) “BMNH(E) / #606199”; c) yellow paper, “*Apterocyclus* / *munroi* m /Sharp, 1908 / PARALECTOTYPE / des. M.J. Paulsen”; d) “*Apterocyclus* / *munroi* / Sharp, 1908 / det. M.J. Paulsen 2012”. Paralectotype male (NHM) labeled: a) handwritten, on cork “*A. munroi* 3rd Typ. D.S.”/ Kaula, Kauai VII’97 / Munro; b) “Sharp Coll. / 1905-313”; c) “BMNH(E) / #606198”; c-d) as above.
Apterocyclus adpropinquans Sharp in Sharp and Scott 1908: 404, **syn. n.** Type material: Holotype male of *A. adpropinquans* (NHM) labeled: a) handwritten, on cork “*A. Adpropinquans* typ. / D.S. / Makaweli Kauai / Perkins”; b) “[260] Makaweli / Kauai [2-3000] ft. / Perkins VI-1894.”; c) “Sandwich Is. / 1912-215”; d) red-bordered circular label “Type”; d) red paper, “*Apterocyclus* / *adpropinquans* ♂ /Sharp, 1908 / HOLOTYPE / des. M.J. Paulsen”; e) “*Apterocyclus* / *munroi* / Sharp, 1908 / det. M.J. Paulsen 2012”.

#### Diagnosis.

**Figures 5–6.**
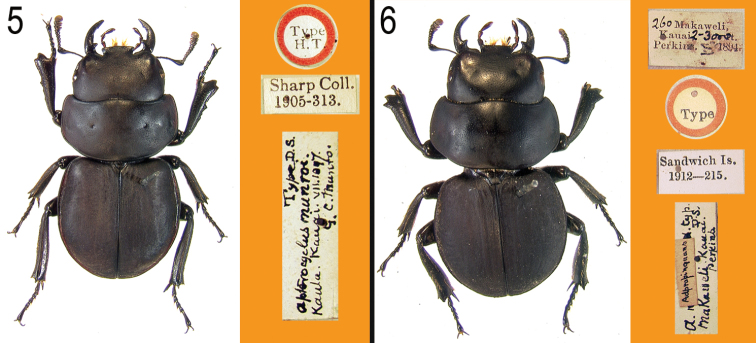
Primary types of *Apterocyclus munroi* and its synonyms, with labels. **5** *Apterocyclus munroi* Sharp, lectotype **6** *Apterocyclus adpropinquans* Sharp, holotype.

Mizunuma (2000) indicated that he considered the synonymy with *Apterocyclus honoluluensis* to be incorrect based on the dentate protibiae that are more similar to those of *Apterocyclus waterhousei*, and that the species might be distinct. We agree with this hypothesis. The protibiae of *Apterocyclus munroi* are externally toothed giving a unique bidentate appearance to the apex (Fig. 5), but the basal two-thirds of the external margin varies from weakly serrate to more or less entire (as in the holotype of *Apterocyclus adpropinquans*, Fig. 6). The protibiae of *Apterocyclus waterhousei* are much wider throughout their length. The scattered long setae on the elytra, especially near the apex, and projected, apically expanded clypeus will readily distinguish *Apterocyclus munroi* from the remaining species.

#### Distribution.

Sharp (1908) described *Apterocyclus munroi* from specimens collected “above Kaula” in 1897, but the identity of this locality is not clear. The holotype of the synonym *Apterocyclus adpropinquans* is reported to be from Makaweli at 2-3000’. No recent specimens with more complete locality data have been studied.

#### Remarks.

Sharp (1908) described *Apterocyclus munroi* from four specimens, although only three are now present in the NHM. The specimen later labeled as the type is here designated as the lectotype. No female specimens have been examined. This and the other names proposed by Sharp (1908) in the *Fauna Hawaiiensis* are attributable to him alone. Although the publication lists D. Sharp and Hugh Scott as the authors of the Coleoptera section, the text for which Scott is responsible is clearly indicated by a superscript (e.g. Discolomidae (sic), p. 431) or is followed by his initials (p. 459). Thus the species have been indicated as being authored by Sharp in Sharp and Scott 1908.

### Apterocyclus palmatus

Taxon classificationAnimaliaColeopteraLucanidae

Van Dyke, 1921

Figs 7 , 15 , 20

Apterocyclus palmatus Van Dyke, 1921: 47, **new status.** Type material: Holotype male (CASC) labeled: a) handwritten “Kauai I. / Hawii (sic) / Alt. 4000 ft. / V-3-1919”; b) “Van Dyke / Collection”; c) handwritten “*Apterocyclus* / *honoluluensis* / *var. palmatus* / Van Dyke / Type”; d) “California Academy / of Sciences / Type / No. [3316]”. One male paratype (CASC) labeled as holotype *a, b, c* [but with ‘Paratype’]. One male paratype (BPBM) labeled a) “[4000 ft] Kauai / [4/28 - 19 Nº5]”; b) “J. A. Kusche / Coll”; c) “[dissected / 10.X.21] / W.M. Giffard / [Nº 5]”; d) handwritten “*Apterocyclus* / *honoluluensis* / *var. palmatus* / Van Dyke / Paratype”; e) on red paper “No. 24642 / Hawaiian Coll. / BISHOP Museum”. One male paratype (BPBM) labeled a) “[4000 ft] Kauai / [4/28 - 19 Nº5]”; b) “J. A. Kusche / Coll”; c) handwritten “*Apterocyclus* / *honoluluensis* / *var. palmatus* / Van Dyke / Paratype”; d) on red paper “No. 24643 / Hawaiian Coll. / BISHOP Museum”. One male paratype (BPBM) labeled a) handwritten “Kauai I. / Hawii (sic) / Alt. 4000 ft. / V-3-1919”; b) handwritten “*Apterocyclus* / *honoluluensis* / *var. palmatus* / Van Dyke / Paratype”; c) handwritten “for / Mr. Giffard”; d) on red paper “No. 24644 / Hawaiian Coll. / BISHOP Museum”.

#### Diagnosis.

**Figures 7–8.**
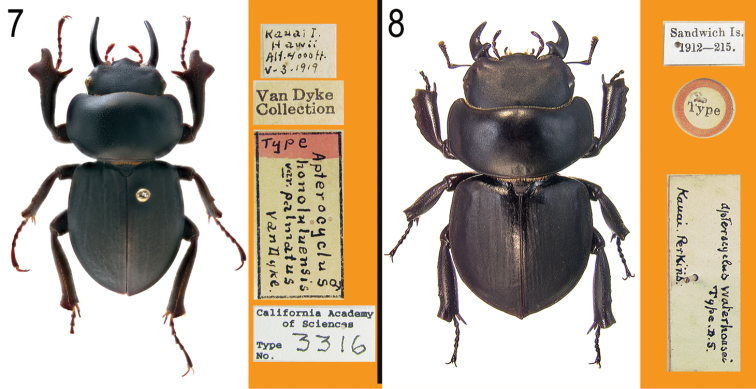
Primary types of *Apterocyclus* species. **7** *Apterocyclus palmatus* Van Dyke, holotype **8** *Apterocyclus waterhousei* Sharp, holotype.

The male mandibles are tusk-like, elongate, nearly twice as long as in any other species, and lack an internal tooth. Also diagnostic are the protibiae that are greatly, triangularly expanded at the apex (Fig. 7), and the projecting tridentate clypeus. The specimens studied are 22–23 mm in length.

#### Distribution.

The species locality is unknown; the only available data is that the specimens were collected at an elevation of 4000 feet on Kauai by Mr. Kusche in 1919.

#### Remarks.

Van Dyke (1922) examined five male specimens, and all were located. Female specimens are not known, and the species has not been collected recently. The clearest evidence of Van Dyke’s overly conservative approach to the genus is that he considered this taxon to be an infraspecific form of *Apterocyclus honoluluensis*; it is arguably the most drastically distinct subspecies of stag beetle ever proposed.

### Apterocyclus waterhousei

Taxon classificationAnimaliaColeopteraLucanidae

Sharp, 1908

Figs 8 , 16 , 21

Apterocyclus waterhousei Sharp in Sharp and Scott 1908: 403. Type material: Holotype male (NHM) labeled: a) handwritten, on cork “*Apterocyclus waterhousei* / Type D.S./ Kauai, Perkins”; b) “Sandwich Is. / 1912–215”; c) red-bordered circular label “Type”; d) red paper, “*Apterocyclus* / *waterhousei* m /Sharp, 1908 / HOLOTYPE / des. M.J. Paulsen”; e) “*Apterocyclus* / *waterhousei* / Sharp, 1908 / det. M.J. Paulsen 2012”.

#### Diagnosis.

The large, median tooth present on the meso- and metatibiae mentioned by Sharp (1908) for the holotype is present on all other specimens of both sexes, and not found in any other species. These large teeth, in addition to the broad, serrate protibiae and almost obsolete canthus separate this species from *Apterocyclus honoluluensis* (a few *Apterocyclus honoluluensis* have a weakly indicated tooth on the mesotibiae). The species is most similar to *Apterocyclus munroi*, but lacks distinct setae on the elytra and the anteriorly divergent clypeus. Specimens range from 18 to 22 mm in length.

#### Distribution.

Specimens have been examined from Kohua/Mohiki [Mohihi?] Stream, Kaholuamano, and near Waialae River (BPBM). Mizunuma (2000) illustrated specimens from Po’omau Canyon, and Fujita (2010) included specimens from Kohua Ridge and Po’omau Canyon.

#### Remarks.

Described from a single male, Sharp (1908) noted the thick legs, ‘crenulate’ protibiae, and pointed elytral apices (Fig. 8). No other specimens have been seen that exhibit the pointed elytral apices of the holotype, and we consider the character to be possibly aberrant and not of specific-level importance.

### Apterocyclus kawaii

Taxon classificationAnimaliaColeopteraLucanidae

Paulsen & Hawks sp. n.

http://zoobank.org/73AD6671-F108-4C0F-840E-28FDD94A4842

Apterocyclus kawaii Paulsen & Hawks, new species. Type material: Holotype male (BPBM) labeled: a) “HAWAIIAN ISLANDS / Kauai I. [Makaweli] / [19.VIII.1978 / RCA Rice”; b) red paper “No. 24714 / Hawaiian Coll. / BISHOP museum”; c) red paper, “*Apterocyclus* / *kawaii* ♂ /Paulsen & Hawks 2014 / HOLOTYPE”. **Type locality:** USA, Hawaii, Kauai Co., Makaweli. Paratype male (MJPC) labeled: a) “Hawaii: Kauai / Robinson Plant Preserve / 21.V.1996 / J.C. Abbott #494; b) black-bordered “[*Apterocyclus* / *honoluluensis* Wat.] / Det. John C. Abbott / [1996]; c) yellow paper, “*Apterocyclus* / *kawaii* ♂ / Paulsen & Hawks 2014 / PARATYPE”.

#### Diagnosis.

**Figures 9–11.**
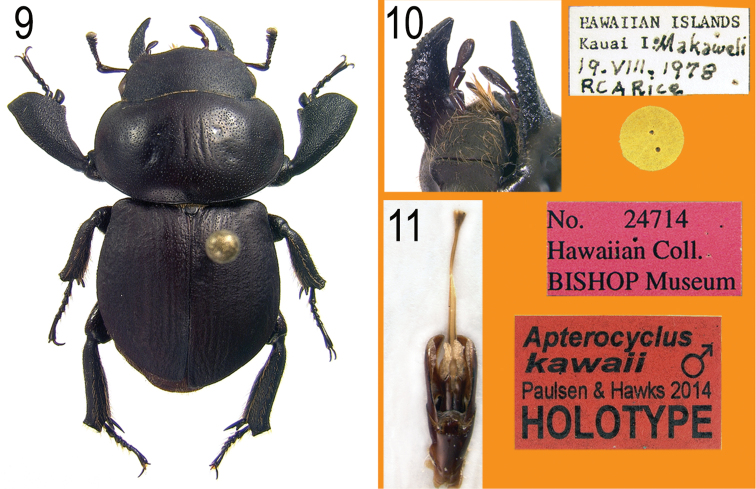
**9** Holotype of *Apterocyclus kawaii*, sp. n., with labels. **10** Mandibles, oblique ventral view showing tubercles **11** Male genitalia.

This distinctive species (Fig. 9) can be immediately recognized by the tubercles present on the ventral surface of the mandibles (Fig. 10). These are not found in any of the described species. Also, the greatly expanded shape, concavity, and rugosity of the protibiae are distinctive (Fig. 9). All other *Apterocyclus* specimens examined have sparsely punctate protibial surfaces, and only *Apterocyclus palmatus* also possesses an internal tooth on the protibia.

**Figures 12–21.**
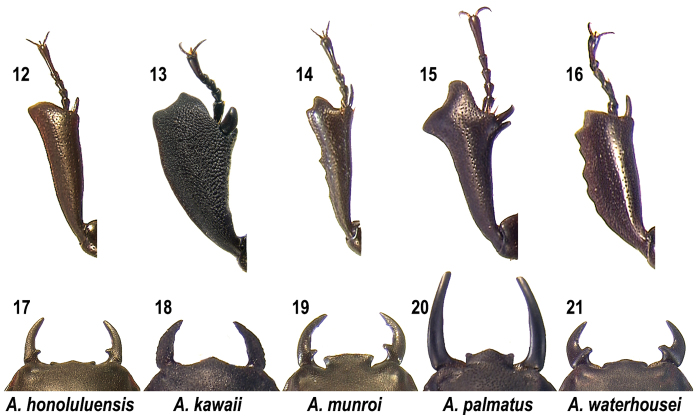
Left protibia and mandibles of *Apterocyclus* species. **12, 17** *Apterocyclus honoluluensis* **13, 18** *Apterocyclus kawaii* **14, 19** *Apterocyclus munroi* **15, 20** *Apterocyclus palmatus* **16, 21** *Apterocyclus waterhousei*.

#### Description, holotype.

*Length*: 16.5 mm. *Width*: 8.2 mm (pronotum). *Color*: Black. *Head*: Surface granulate and with scattered moderate punctures. Eyes with ocular canthus almost obsolete. Antennal club small, short (shorter than scape), antennomeres 8 and 9 of club tomentose only distally, visible surfaces of antennomere 10 more or less entirely tomentose. Clypeofrontal area tumid. Clypeus short, not projecting, broadly triangular with pointed apex. Mandibles short, simply falcate (lacking internal tooth, but with irregular internal margin), broadly flattened; surface externally and ventrally with numerous small tubercles (Fig. 10). Mentum semicircular, surface granulate, setose. *Pronotum*: Form short, wider than elytra, broadly rounded laterally (posterior angles obsolete). Surface shiny on disk with moderately deep punctures, becoming granulate near angles. *Elytra*: Form almost circular, as wide together as long. Surface alutaceous but weakly shiny with moderate, shallow punctures, some in vague rows. *Legs*: Protibiae concave ventrally, widened apically, surface densely, rugosely punctate; external margin with one large tooth near apex. Apical spur short, spatulate. Meso- and metatibiae robust, lacking external teeth. *Abdomen*: Male genitalia (Fig. 11) with permanently everted internal sac flared at apex (as in congeners).

#### Paratype variation.

Length: 22.9 mm. Width: 10.8 mm. *Head*: Clypeofrontal area indented; clypeus with apex less acute.

#### Etymology.

The species is named for our colleague Shinya Kawai, of Tokyo, Japan, in honor of his work on Lucanidae, especially on the similarly flightless genus *Colophon* Gray of South Africa, and in gratitude for the assistance he has provided to MJP in both research and field collecting in Japan. The specific epithet is a noun in the genitive case.

#### Distribution.

Two specimens are known, both males, from the Makaweli [Kaumakani] area.

#### Remarks.

Nothing is known of the life history of this species. Although published as a rediscovery of *Apterocyclus honoluluensis*, the specimen discussed by Abbott and Petr (1997) is the paratype of this new species. The specimen was collected at dusk on a footpath along a ridge at 1000m (Abbott and Petr 1997), although the specimen is damaged and appears to have been stepped upon.

### Key to the recent species of *Apterocyclus* Waterhouse*

**Table:** 

1	Mandibles ventrally tuberculate (Fig. 10); protibia broad and densely, rugosely punctate dorsally (Fig. 13)	*Apterocyclus kawaii* Paulsen & Hawks, n. sp.
1’	Mandibles simply punctate, not tuberculate; protibia simply and sparsely punctate dorsally	2
2	Ocular canthus distinct. Exterior margin of protibia usually entire, not strongly dentate (Fig. 12)	*Apterocyclus honoluluensis* Waterhouse
2’	Ocular canthus obsolete. Exterior margin of protibia dentate with at least 1 external tooth after apex (Figs 14–16)	3
3	Protibia with 1 large external and 1 large internal tooth (Fig. 15); mandibles longer than head, lacking internal tooth (Fig. 20)	*Apterocyclus palmatus* Van Dyke
3’	Protibia externally weakly to distinctly serrate, without internal tooth; mandibles shorter than head, with internal tooth (Figs 19, 21)	4
4	Elytra with distinct setae near apex. Meso- and metatibiae unarmed	*Apterocyclus munroi* Sharp
4’	Elytra with setae indistinct. Meso- and metatibiae with distinct external median tooth	*Apterocyclus waterhousei* Sharp

* Females are known only for *Apterocyclus honoluluensis* and *Apterocyclus waterhousei*. In these two species the protibial form is identical in both sexes. This may not be true for *Apterocyclus kawaii* and *Apterocyclus palmatus* given the more distinctive male protibiae. Females that lack ocular canthi should be tentatively identified until the females of all species are known.

## Revised catalog

***APTEROCYCLUS* Waterhouse 1871: 315**

***Apterocyclus honoluluensis* Waterhouse** 1871: 315

*Apterocyclus deceptor* Sharp in Sharp and Scott 1908: 405, synonym.

*Apterocyclus feminalis* Sharp in Sharp and Scott 1908: 405, synonym.

*Apterocyclus varians* Sharp in Sharp and Scott 1908: 404, synonym.

***Apterocyclus kawaii* Paulsen & Hawks**, new species.

***Apterocyclus munroi* Sharp** in Sharp and Scott 1908: 403, revised status.

*Apterocyclus adpropinquans* Sharp in Sharp and Scott 1908: 404, new synonymy.

***Apterocyclus palmatus* Van Dyke** 1921: 47, revised status.

***Apterocyclus waterhousei* Sharp** in Sharp and Scott 1908: 403.

## Supplementary Material

XML Treatment for Apterocyclus honoluluensis

XML Treatment for Apterocyclus munroi

XML Treatment for Apterocyclus palmatus

XML Treatment for Apterocyclus waterhousei

XML Treatment for Apterocyclus kawaii
